# IDH-mutant astrocytoma with EGFR amplification—Genomic profiling in four cases and review of literature

**DOI:** 10.1093/noajnl/vdac067

**Published:** 2022-05-10

**Authors:** Melissa Umphlett, Khawaja Hasan Bilal, Michael L Martini, Abigail K Suwala, Sadhna Ahuja, Omid Rashidipour, Isabelle Germano, Matija Snuderl, Peter Morgenstern, Nadejda M Tsankova

**Affiliations:** Department of Pathology, Molecular and Cell-Based Medicine, Icahn School of Medicine at Mount Sinai, New York, New York, USA; Department of Pathology, Mount Sinai West, New York, New York, USA; Department of Pathology, Mount Sinai West, New York, New York, USA; Department of Neurosurgery, Icahn School of Medicine at Mount Sinai, New York, New York, USA; Department of Neuropathology, Institute of Pathology, Heidelberg University Hospital, Heidelberg, Germany; Clinical Cooperation Unit Neuropathology, German Cancer Research Center (DKFZ), Heidelberg, Germany; Consortium for Translational Cancer Research (DKTK), Heidelberg, Germany; Department of Neurological Surgery, Helen Diller Research Center, University of California San Francisco, San Francisco, California, USA; Department of Pathology, Molecular and Cell-Based Medicine, Icahn School of Medicine at Mount Sinai, New York, New York, USA; Department of Pathology, Molecular and Cell-Based Medicine, Icahn School of Medicine at Mount Sinai, New York, New York, USA; Department of Neurosurgery, Icahn School of Medicine at Mount Sinai, New York, New York, USA; Department of Pathology, NYU Langone Medical Health, New York, New York, USA; Department of Neurosurgery, Icahn School of Medicine at Mount Sinai, New York, New York, USA; Department of Pathology, Molecular and Cell-Based Medicine, Icahn School of Medicine at Mount Sinai, New York, New York, USA

IDH-mutant astrocytomas carry significantly better prognosis compared to their IDH-wildtype grade 4 (glioblastoma) counterpart. Several molecular diagnostic markers have emerged in the 2021 CNS WHO classification, with powerful prognostic implications to consider when classifying diffuse IDH-mutant astrocytomas.^1–3^*EGFR* amplification status, associated with aggressive glioma behavior^4^ and now regarded as a molecular feature of glioblastoma, is not currently a diagnostic consideration in IDH-mutant astrocytomas.^1^ Although uncommon, IDH-mutant astrocytomas with *EGFR* amplification exist in large published datasets but their relevance has been under-emphasized and remains poorly understood.^5–8^

In an effort to better understand the biology of “IDH-mutant astrocytoma with *EGFR* amplification,” we present the clinical and molecular profiles in four such rare cases encountered at two institutions; evaluate them based on cytogenetics, DNA sequencing, and DNA methylation profiling; and scrutinize published datasets for this specific entity to gain further insight into its diagnostic and prognostic implications.

## Clinical Presentation

**Figure 1.**
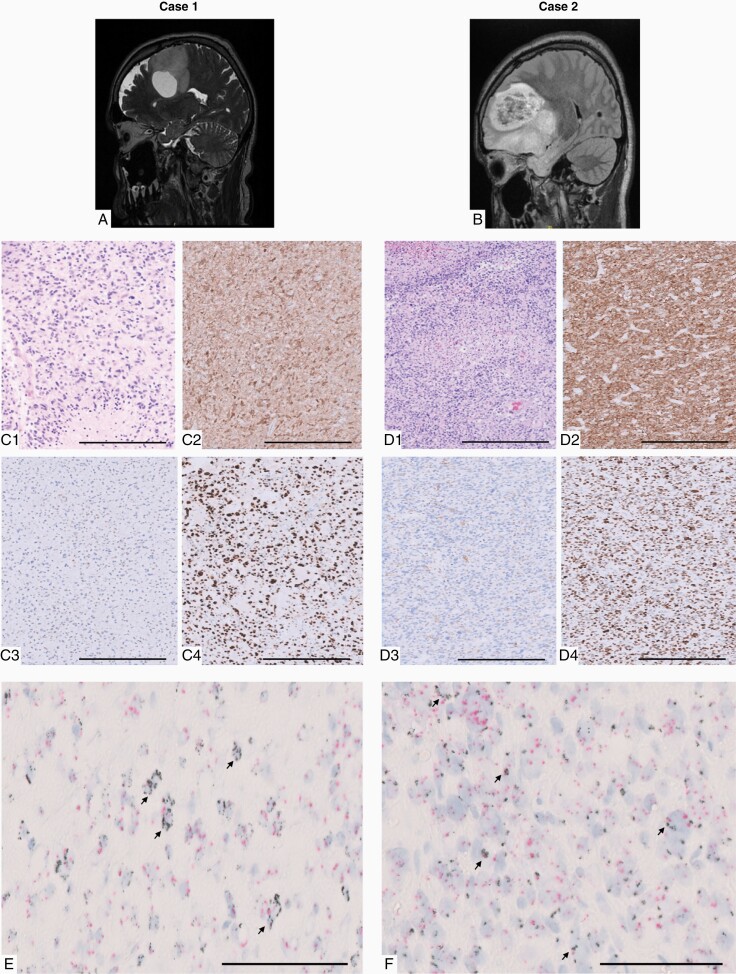
Radiographic and histologic findings of cases 1 and 2. (A and B) MR imaging of tumors at initial presentation—sagittal T2 CUBE (A); sagittal flair (B). (C and D) Histological evaluation. C1, D1—infiltrative glial neoplasm with moderate nuclear atypia and focal palisading necrosis (H&E, 200×). C2, D2—diffuse expression of mutant IDH1R132H protein in tumor cells by immunohistochemistry (200×). C3, D3—loss of nuclear ATRX protein expression in tumor cells by immunohistochemistry (200×). C4, D4—strong nuclear positivity for TP53 by immunohistochemistry (200×). Scale bar = 100 µm. (E and F) EGFR amplification detected in tumor cells using multiplex dual color DNA probe in situ hybridization (indicated by arrows). EGFR probe is black; CEP7 probe is red; ISH EGFR gene/CEP7 ratio of >2.0 (600×). Arrows point to cells with amplification. Scale bar = 50 µm.

We report four cases encountered at two institutions between 2015 and 2020, diagnosed as “Glioblastoma (Astrocytoma), IDH-mutant, WHO grade 4,” found to carry *EGFR* amplification, an alteration diagnostic of IDH-wildtype Glioblastoma.^1^ Three patients were adults and one was pediatric. All presented with large and infiltrative, heterogeneously enhancing MRI lesions (Figure 1A and B), and underwent gross total tumor resections followed by chemoradiation therapy. Case 1, a 59-year-old man with a large cystic right frontal mass, underwent resection and was discharged from the hospital on postoperative day 5. He passed away 16 days after his initial surgery. Case 2, a 15-year-old boy with a left frontal tumor, underwent sub-total resection and received standard therapy of Temozolomide and proton beam therapy along with Optune device. He had recurrent disease and progression through therapy 16 months after surgery, which was initially treated with Bevacizumab 17 months after surgery. He continued to progress and died of his disease 20 months after his initial resection. Case 3, a 28-year-old man with Charcot Marie Tooth syndrome and left frontoparietal tumor, was lost to follow-up a year after initial resection and Stupp-protocol treatment. Case 4, a 37-year-old man with a left parietal tumor, recurred a year after treatment with Procarbazine with 1-(2-chloroethyl)-3-cyclohexyl-1-nitrosourea and adjuvant radiation, and is in stable condition at his most recent follow-up, 4 years after initial presentation.

## Pathological Evaluation

Histologic examination in all cases revealed moderate-to-highly cellular, diffusely infiltrative glial neoplasms composed of pleomorphic glioma cells with astrocytic appearance, including angulated nuclei, fibrillary cytoplasm, and occasional gemistocytic morphology (Figure 1C1 and D1). Nuclear atypia was consistently seen and mitotic figures were readily apparent with proliferation index ranging between 10% and 40%. Grade 4 histological features, including microvascular proliferation and palisading-type tumor necrosis, were present in all cases (Figure 1C1 and D1). In immunohistochemical studies, cases showed diffuse positivity for GFAP, mutant IDH1R132H expression (Figure 1C2 and D2), nuclear loss of ATRX (Figure 1C3 and D3), and strong nuclear P53 (Figure 1C4 and D4), suggestive of mutant *IDH1R123H*, *ATRX*, and *TP53* status, respectively. None of the cases were positive for mutant BRAFV600E or H3K27M. As part of routine clinical workup, analysis for *EGFR* amplification was performed using multiplex dual color DNA probe chromogenic in situ hybridization (CISH) or fluorescent in situ hybridization (FISH). These assays identified focal and/or diffuse amplification of *EGFR* at chromosome 7 in all cases: case 1 with 10 average EGFR/CEP7 CISH signal ratio (Figure 1E); case 2 with 5 average EGFR/CEP7 CISH signal ratio (Figure 1F); case 3 with 2.1 FISH ratio and 5 *EGFR* copies on average; and case 4 with 3.6 FISH ratio and 9.7 *EGFR* copies on average.

## Molecular Studies

Additional molecular studies were performed to further profile all cases and confirm histological findings, including clinically validated targeted next-generation DNA sequencing (NGS) (Sema4 or FoundationOne Laboratories, cases 1 and 2), and DNA methylation plus cytogenetics profiling for large copy number alterations (NYU, all cases). Targeted DNA sequencing confirmed *IDH1*, *TP53*, and *ATRX* mutations, and uncovered *PIK3CA* mutations in case 1 and case 2 (Table 1). Cytogenetic profiling confirmed *EGFR* amplification in all cases and detected additional copy number alterations, including gains at *PDGFRA* (case 1), *MYC* (case 1), *FGFR1/TACC1* (case 1), *TERT* (case 2); and losses at *CDKN2A/B* (case 1, case 2), *PTEN* (case 1, case 2), *CDK4* (case 1), *MDM2* (case 1), *RB1* (case 1), *C19MC* (case 1, case 2), *MGMT* (case 1, case 2), *MYB* and *MYBL1* (case 2), *MYC* (case 2), and *NF2* (case 2) (Table 1, Supplementary Figure 1). Furthermore, *MGMT* promoter methylation was detected by pyrosequencing in case 1 and case 2, and it was not detected in case 3 and case 4 (Table 1).

**Table 1.** Summary of IDH-mutant EGFR-amplified Glioma Features From Literature Review

Citation	EGFRamp+ IDH-mut/All IDH-mut Gliomas (Grade)	DNA Methylation Status	Other Molecular Findings in EGFRamp+ IDH-mut Gliomas (Number of Cases)	Age Range (Sex)
Li et al^7^	7/57 (grade 4)	3 G-CIMP-high 4 G-CIMP-low	SNV: *TERT* (2) G: *CCND2* (2), *PDGFRA* (1), *CDK4* (1), *MYC* (1) L: *PTEN* (1), *CDKN2A* (2), *RB1* (2), *NF2* (1)	4 > 40 years old 3 < 40 years old (4 F, 3 M)
Bai et al^8^	16/82 (grades 2-3)			
Brennan et al^5^	5/30 (grade 4)	5 G-CIMP-high	SNV: *TP53* (4), *ATRX* (2) G: *MYC* (2) L: *CDKN2A/B* (2), *NF1* (1)	5 < 40 years old
Verhaak et al^6^	1/12 (grade 4)		SNV: *EGFR* (1) G: *PDGFRA* (1) L: *CDKN2A/B* (1)	
Umphlett et al (current study)	2/56 (Grade 4) (MSSM)	2 *MGMT* methylated 2 *MGMT* unmethylated	**SNV:** *TP53* (4), *ATRX* (2), *PIK3CA* (2) **G:***TERT* (1), *PDGFRA* (1), *MYC* (1), *FGFR1/TACC1* (1) L: *CDKN2A/B* (2), *PTEN* (2), *C19MC* (2), *MGMT* (2), *MYB* (1), *MYBL1* (1), *MYC* (1), *NF2* (1), *CDK4* (1), *MDM2* (1), *RB1* (1), *MTAP* (1)	3 < 40 years old 1 > 40 years old (All M)

Abbreviations: EGFRamp+, EGFR amplification; G, gain; G-CIMP-high, glioma-CpG island methylator phenotype high; G-CIMP-low, glioma-CpG island methylator phenotype low; IDH-mut, IDH-mutant; L, loss; SNV, single nucleotide variant.

The presence of both *EGFR* amplification and *IDH* mutation in these high-grade astrocytoma tumors was unusual and raised questions within our clinical team in regards to the tumors’ correct nomenclature, biological behavior, possible syndromic genomic instability, and, ultimately, patient prognosis.

## DNA Methylation-Based Tumor Classification

**Figure 2.**
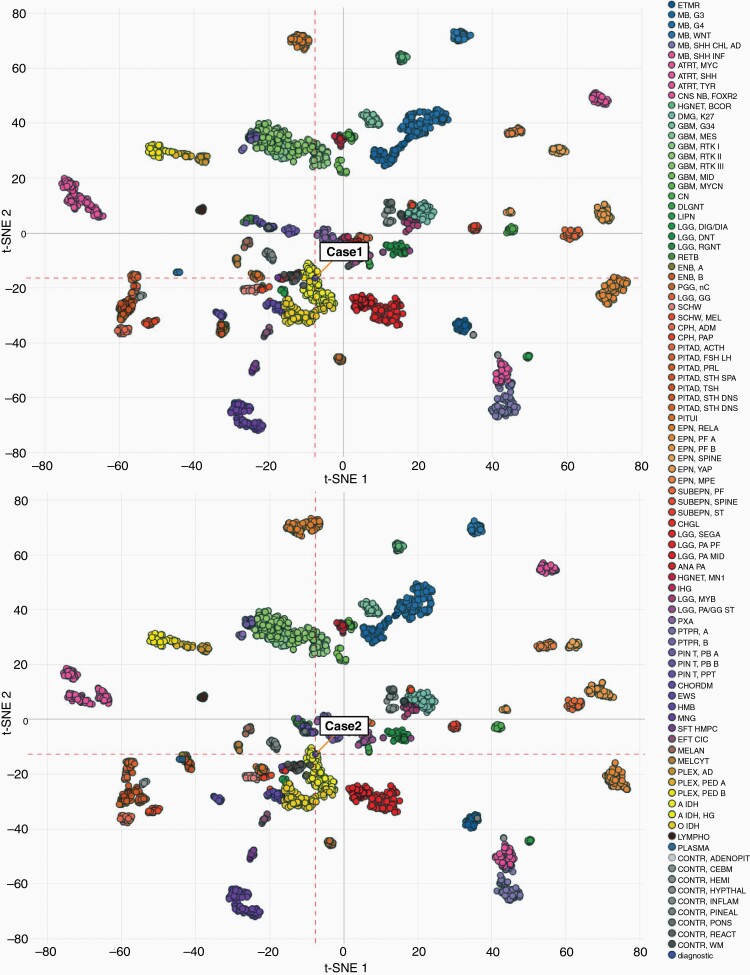
DNA methylation-based tumor classification. Dimensionality reduction (t-SNE) shows unbiased clustering of case 1 and case 2 within the “IDH-mutant High-Grade Astrocytoma” classifier group (all cases displayed similar clustering pattern).

To independently validate histological diagnoses and NGS findings, as well as to explore further the relationship of *EGFR*-amplified IDH-mutant astrocytomas to other CNS tumors, we performed clinically validated NYU whole-genome DNA methylation profiling using Illumina EPIC array and analyzed using the Heidelberg brain tumor classifier.^9^ Notably, all four cases were classified as “IDH-mutant High-Grade Astrocytoma” with high confidence scores (0.99 0.997, 0.98, and 0.99; cases 1-4, respectively). confirming that IDH mutation-induced hypermethylation remains preserved in these tumors. Reduced dimensionality (t-SNE) visualization corroborated unbiased clustering of these cases within the “IDH-mutant High-Grade Astrocytoma” group and away from all IDH-wildtype GBM groups (“GBM, RTK I”; “GBM RTK II”; “GBM RTK III”; “GBM-MYCN”; “GBM-MES”; “GBM-MID”; “GBM-K27”; “GBM-G34”) (Figure 2).

We considered the possibility that excessive genomic instability may have led to *EGFR* amplification in our cases and therefore also tested the four cases on a classifier that includes primary mismatch repair-deficient IDH-mutant astrocytomas (PMMRDIA) as a separate category. PMMRDIA encompasses a small subset of IDH-mutant astrocytomas, which have hereditary mismatch repair (MMR) deficiency and worse clinical outcome.^10^ Importantly, none of the four cases clustered with “PMMRDIA IDH-mutant astrocytomas” (data not shown). Furthermore, DNA methylation-based prediction of the MLH1 gene status, one of the most commonly mutated DNA MMR complex members, as well as immunohistochemical studies for loss of expression of MLH1, MSH2, MSH6, and PMS2 MMR proteins, did not reveal evidence for microsatellite instability (data not shown). Overall, despite the presence of *EGFR* amplification in all four cases, a diagnostic molecular marker in IDH-wildtype glioblastoma and a predictor of its aggressive behavior, IDH-mutant status was the determinate factor for an epigenetics-based classification of these high-grade astrocytomas.

## Literature Review

Limited by long-term survival data in our prospective cases, we performed retrospective review of the literature to explore further the prognostic significance of *EGFR* amplification within IDH-mutant astrocytomas. No studies were found specifically focused on *EGFR*-amplified IDH-mutant astrocytomas. Thus, we focused on large glioma dataset studies, searching for the specific co-occurrence of *IDH* mutation and *EGFR* amplification.^5–8^

In the Li et al’s study examining 57 IDH-mutant GBM, 7 (12.3%) had *EGFR* amplification.^7^ Of these, only three cases (40%) had a glioma methylator (G-CIMP) high phenotype typically found in IDH-mutant grade 4 tumors with more favorable prognosis (Table 1). Other copy number alterations within this series included deletion of *CDKN2A* and amplification of *CCND2*, *PDGFRA*, *MYC*, *CDK4*, and *MET* (Table 1). All *MET*-amplified tumors belonged to the G-CIMP-low subgroup and exhibited *CDKN2A* alterations. Importantly, worse overall survival (OS) was associated within the group of tumors with G-CIMP-low, *CDKN2A* deletion, and *MET* amplification status (median OS of 252 days). In the Brennan et al’s study profiling 332 GBM tumors, 5 out of 30 IDH-mutant GBM cases were identified to be *EGFR*-amplified (16.7%), all of which were G-CIMP-high, with 2 out of 5 containing MYC amplification and/or *CDKN2A/B* loss.^5^ (Table 1). Only one of the five cases was associated with lower OS (<9 months).^5^ In the Verhaak et al’s study, 1 out of 12 (8.3%) IDH-mutant GBM cases had co-occurrence of *EGFR* and *PDGFRA* amplifications with *CDKN2A/B* loss, with unclear prognostic significance^6^ (Table 1). Finally, in the Bai et al’s study, 16 out of 86 IDH-mutant grade 2-3 gliomas were *EGFR*-amplified (18.6%)^8^ (Table 1). Although *EGFR* status showed statistically significant lower survival within grade 2-3 tumors, *MYC* amplification, *PTEN* loss, and *CDKN2A* loss status were associated with tumor progression.^8^ Generally, none of the studies found *EGFR* amplification to be an independent marker of worse OS within grade 4 IDH-mutant astrocytomas, unless it co-occurs with *CDKN2A/B* loss, *MET* amplification, and/or G-CIMP-low status.

## Discussion/Conclusion

Identifying prognostically relevant subgroups of astrocytic gliomas is extremely important for clinical trial inclusion and patient care. *EGFR* amplification has long been established as an independent and significant factor associated with shorter survival time and poorer prognosis in patients with glioblastoma, and therefore its co-occurrence in the setting of IDH-mutant astrocytomas warrants closer examination.^4^ In the four cases presented, the co-existence of both alterations challenged the diagnostic and prognostic stratification of these tumors, eliciting uncertainty as to the tumors’ clinical behavior.

Reports of IDH-mutant astrocytomas with *EGFR* amplification are rare in the literature.^5,8,11^ Indeed, at one of the authors’ institutions, only two such cases were detected out of 56 IDH-mutant astrocytomas between 2019 and 2020. Consequently, the clinical relevance of *EGFR* amplification in IDH-mutant astrocytomas has not been established. In the most recent CNS WHO classification, mutant IDH is a diagnostic molecular marker of diffuse astrocytomas, whereas *EGFR* amplification is a diagnostic molecular marker of glioblastoma.^1–3^ In cIMPACT-NOW update 5, many molecular alterations were considered to identify IDH-mutant astrocytomas with a clinical course corresponding to WHO grade 4, including *CDKN2A/B* homozygous deletion, *CDK4* amplification, *RB1* mutation, or homozygous deletion, *PIK3CA* or *PIK3R1* mutations, *PDGFRA* amplification, *MYCN* amplification, global DNA methylation levels, chromosome 14 loss, and genomic instability.^3^ However, *EGFR* amplification was not included in this evaluation.

Our literature review analysis establishes that IDH-mutant astrocytomas with *EGFR* amplification are not as rare as previously considered. By mining several large glioma datasets for this specific subtype, we uncovered a relatively high occurrence of *EGFR* amplification/copy number gain in IDH-mutant astrocytomas, 8%-19% depending on the study.^5–8^ The discrepancy between the rare clinical recognition of these tumors and their more frequent appearance in published datasets may be related to the lack of additional molecular testing in astrocytomas, once they are diagnosed as IDH-mutant using immunohistochemistry.

DNA methylation-based classification analysis in our case series showed consistent clustering of tumors based on IDH-mutant status rather than on *EGFR* amplification, indicating that these tumors should be considered as “IDH-mutant astrocytomas” for diagnostic purposes, despite the presence of *EGFR* amplification. Furthermore, albeit limited to a single study, current literature suggests that *EGFR* amplification in grade 4 IDH-mutant astrocytomas is not associated with worse OS, unless *CDKN2A/B* loss is also detected.^7^ This is consistent with current CNS WHO recommendations, which recently established homozygous loss of *CDKN2A/B* as an independent marker of poor prognosis within IDH-mutant astrocytomas, sufficient to diagnose a tumor as CNS WHO grade 4 in the absence of microvascular proliferation and tumor necrosis.^1,12^ It also correlates with the poor outcome observed in case 1 and case 2, both of which displayed concurrent *CDKN2A/B* loss.

Although none of the cases in our series contained microsatellite instability, co-occurrence of *IDH* and *EGFR* alterations in an astrocytoma may indicate a hypermutated phenotype and/or hereditary MMR deficiency (PMMRDIA). Such tumors have more aggressive clinical behavior but may respond to specific immune checkpoint therapies.^10^ Therefore, the presence of mutant *IDH* plus *EGFR* amplification in a diffuse glioma should alert clinicians to rule out MMR deficiency and/or microsatellite instability. Further investigation is warranted to explore the impact of *EGFR* amplification on the biology and clinical progression of IDH-mutant astrocytomas, including processes, such as tumor growth, invasion, recurrence, and on patients’ response to specific therapeutic regimens. Despite significant preclinical evidence for the therapeutic potential of monoclonal antibodies and tyrosine kinase inhibitors (TKIs) that target EGFR, clinical trials thus far have failed to demonstrate significant survival benefits in patients with glioblastoma, regardless of *EGFR* amplification and mutation status.^13,14^ Ongoing studies continue to explore EGFR amplification status as a potentially targetable marker and our report flags an important subgroup of IDH-mutant astrocytomas that may respond to such therapy if and when it becomes actionable.

## Supplementary Material

vdac067_suppl_Supplementary_Figure_S1
