# Supplementary material for: IDH-mutant astrocytoma with EGFR amplification—Genomic profiling in four cases and review of literature
Source: Neurooncol Adv. 2022 May 10;4(1):vdac067. doi: 10.1093/noajnl/vdac067 (PMC9159664; doi:10.1093/noajnl/vdac067)
Supplement: vdac067_suppl_Supplementary_Figure_S1 [file vdac067_suppl_supplementary_figure_s1.docx]

**Molecular Tumor Board Case Report: IDH-mutant Astrocytoma with EGFR Amplification – Genomic Profiling in Four Cases and Review of Literature**


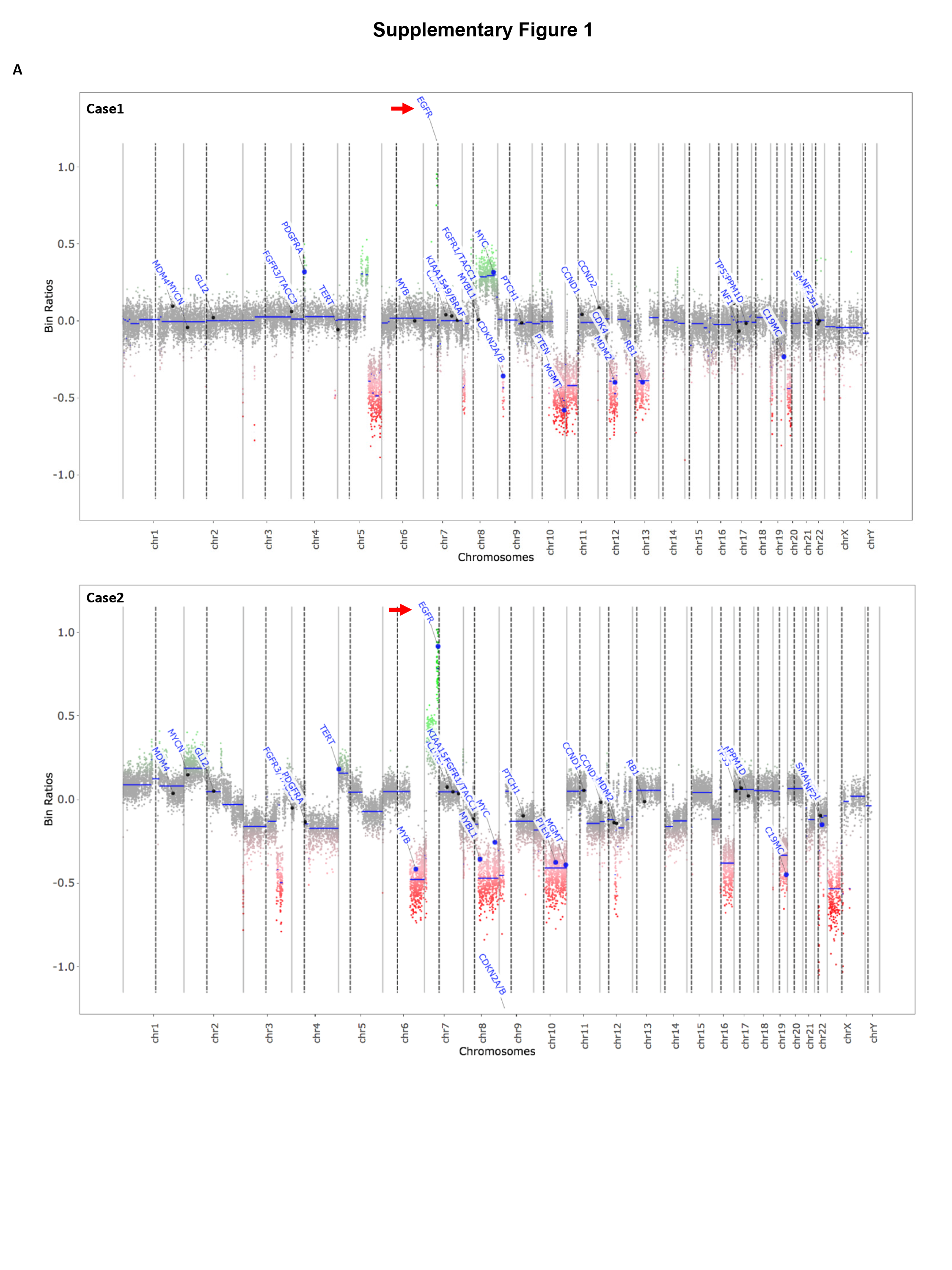


Supplementary Figure 1 **DNA methylation-based cytogenetic profiling**

Cytogenetic profiling in Case1 and Case2 confirms *EGFR* amplification and illustrates additional copy number alterations.
